# Democratic South Africa at 25 – a conceptual framework and narrative review of the social and structural determinants of adolescent health

**DOI:** 10.1186/s12992-021-00679-3

**Published:** 2021-03-29

**Authors:** Tanya Jacobs, Asha George

**Affiliations:** School of Public Health, University of the Western Cape, Bellville, South Africa

**Keywords:** Social and structural determinants, Adolescent health, Gender, Intersectionality, South Africa, Micro, Meso, Macro

## Abstract

Twenty-five years into South Africa’s constitutional democracy provides an opportunity to take stock of the social and structural determinants of adolescent health. Those born in democratic South Africa, commonly known as the ‘Born Frees’, are perceived to be able to realise equal rights and opportunities, yet many factors constrain their lives. In bringing together approaches to understanding context in health policy and systems research and the social determinants of health, the paper develops a conceptual framework to guide the narrative review examining the key contextual social and structural determinants of adolescent health in South Africa. Illustrative examples drawing from 65 papers from public health and the social sciences describe and link these determinants across micro, meso and macro levels of society, their global determinants, and their intersections with compounding axes of power and inequality.

At a micro level individual adolescent sexual and gender identities are expressed through multiple and evolving forms, while they experience growing autonomy and agency, they do so within a broader context characterised by regressive social norms, gender inequality and other intersecting power relationships. At the meso level, organisational and sectoral determinants shape adolescents health and rights, both in being supportive, but they also replicate the biases and inequalities that characterise South African society. In addition, the macro level national and global determinants, such as the structural colonial and apartheid legacies, shape adolescents' health. Despite constitutional and other legislative rights, these determinants and compound economic, geographic, gender and other intersecting inequalities.

A key finding is that current experiences and health of adolescents is shaped by past social and structural determinants and power relations, with apartheid inequalities still echoing in the lives of the adolescents, 25 years into democracy. More research and work is needed to provide insights into determinants of adolescent health beyond just the micro level, but also at the interrelated and dynamic meso and macro levels, nested in global determinants. The findings raise critical considerations and implications for understanding the social and structural determinants in the South African context and what this means for adolescent health in the SDG era.

## Background

Twenty-five years into South Africa’s constitutional democracy provides an opportunity to take stock of the social and structural determinants that shape adolescent health. Those born in democratic South Africa, commonly known as the ‘Born Frees’, are perceived to be able to realise equal rights and opportunities. However, the persistence of past inequalities continues to shape their lives, their health and the systems in which they live.

Adolescents, make up almost a fifth of South Africa’s population [1] and while national school attendance stands at 98% [2], estimates for completion of secondary education are at 49% [3]. Equally of concern is the very high unemployment rate for both youth and adults in South Africa. In the first quarter of 2018, 38% of young people aged 15–34 were unemployed [4]. Also alarming is the level of violence experienced by adolescents in South Africa. Approximately one in three adolescents (35%), reported that they experienced some form of sexual abuse during their lifetime [5, 6]. Gender power relations underlie  the perpetration and experience of violence. In one study, in Cape Town, 10% of boys reported forcing a partner to have sex, while 39% of girls reported physical victimization [7]. Further, for example, in terms of adolescent sexual and reproductive health, 16% of women aged 15–19 years have begun childbearing. Of these only 52% were reported to be attending school, compared to 83% of childless adolescents in 2016 [1]. Despite HIV incidence decreasing from 2012, it remains high, particularly among female youth aged 15–24 years [8]. HIV incidence rates for females aged 15–24 years were three-times that of their male counterparts in 2017.

While these descriptive statistics help illustrate one dimension of adolescent health, it is also important to locate adolescents beyond a public health lens. The comprehensive aims of the Sustainable Development Goals (SDGs), aligned with the United Nations Secretary General’s Global Strategy (2016–2030), emphasises that all women, children and adolescents not only survive, but thrive and transform. This provides a window of opportunity to develop new paradigms for ensuring inclusiveness and responsiveness to the rights and needs of adolescents [9, 10] as significant societal assets whose contributions and meaningful participation are critical for societal wellbeing [10].

**Fig. 1:**
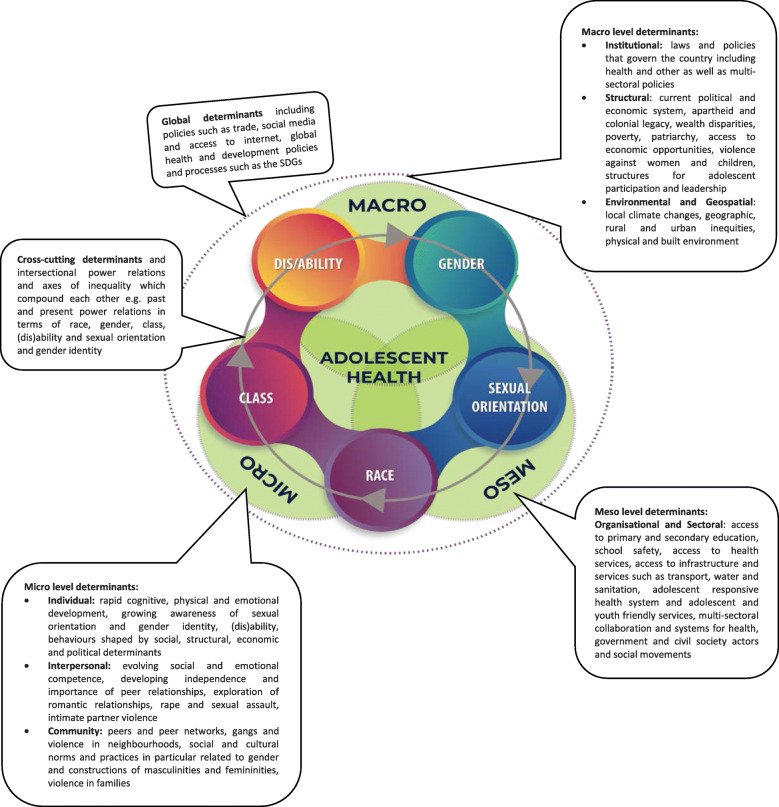
Social and structural determinants of adolescent health in South Africa

The paucity of conceptual frameworks which take into account the social and structural determinants of adolescent health in South Africa, and their dynamic interaction across the micro, meso and macro levels, nested in global contexts, limits the ability of policy makers and practitioners, as well as researchers, to optimally identify their role and contribution to adolescent health. Seizing this window of opportunity, this paper seeks to conceptualise the contextual factors that shape adolescent health, by presenting a conceptual framework (Fig. 1) and narrative review of existing multi-disciplinary literature which describes the social and structural determinants of adolescent health at micro, meso and macro levels, as well as how these intersect with and compound other with axes of power and inequality. In doing so it aims to provide greater understanding of the implications of these contextual determinants shaping adolescent health for South African health systems and policy in the SDG era.

## Methods

The review considers approaches to understanding and conceptualising ‘context’ from Health Systems and Policy Research (HPSR), the Social Sciences, as well as intersectional perspectives, to develop a conceptual framework to map the social and structural determinants of adolescent health in South Africa (Fig. 1). It builds on the WHO Social Determinants of Health Framework [11] and recent publications on adolescent health [12–15], many of which draw on socio-ecological theory [16]. It presents interrelated micro (interpersonal), meso (organisational), and macro (structural) contextual determinants within the lasting legacy of apartheid, 25 years after South Africa’s transition to democracy. In addition, it emphasises cross-cutting past and present social and structural determinants, such as racial and gender inequality. These intersect with and compound other cross-cutting social and structural determinants, such as class, (dis)ability, sexual orientation, and other forms of discrimination and marginalisation to construct and determine the health of adolescents [17]. Importantly, these national determinants are nested in broader global contextual determinants, such as alcohol and tobacco policies, global trade, global health and development policies and processes, such as the SDGs, religious and neo-liberal ideologies, globalisation, migration, war/conflict and climate change, as well as social media and access to the internet.

The paper’s conceptual framework (Fig. 1) guided the search strategy for publications, including grey literature, which were sourced in an iterative manner through systematic internet searches from September 2018 to December 2019. The focus of the searches was on the phenomena of interest i.e. social and structural determinants shaping adolescent health in post-apartheid South Africa. Search terms included a combination of ‘adolescent’, ‘social determinants’, ‘structural determinants’, ‘inequality’, ‘gender, ‘context’, ‘health, ‘macro’, ‘meso’, ‘micro’, ‘global’ ‘South Africa’, ‘intersectionality’. The 65 included publications were sourced from Public Health, but were also intentionally selected from the Social Sciences, to integrate disciplinary perspectives. This included fields such as Sociology, Anthropology, History and Cultural studies, Gender studies, Sexualities studies, Education and Criminal Justice. The conceptual framework was central to our analysis and findings from the thematic analysis were used to further refine it in an iterative manner. Importantly, this paper does not aim to provide a definitive analysis of all the social and structural determinants that shape adolescent health in South Africa, however it foregrounds key insights across disciplines with illustrative examples.

## Findings

### Social and structural determinants: intersecting micro, meso and macro levels

We describe key social and structural determinants shaping adolescent health in South Africa, starting from the micro level through to the macro level as well as the global level, noting that there is dynamic interaction between levels, also shaped by intersectional cross-cutting determinants, as illustrated in Fig. 1.

### Micro level: individual, interpersonal and community

In this section we describe micro level determinants which include the individual, interpersonal and community aspects as presented in the conceptual framework (Fig. 1). We illustrate how gender identities and sexual orientation are shaped by social and cultural norms and practices, influenced by violence in relationships and communities, class inequality, spatial segregation, racism and homophobia.

Adolescence is a time of key developmental transitions towards adulthood with significant cognitive, biological, physical, psychosocial and emotional changes, which can bring both excitement and challenges [10]. The reviewed literature details how the individual experiences and behaviour of adolescents in South Africa is shaped by inequalities and violence at the meso and macro level, as mapped out in the sections below.

In South Africa, adolescent femininities and masculinities are constructed simultaneously by historical processes of cultural and religious integration and contemporary pressures, where there is a resurgence of ‘traditionalism’, in some parts of the country [18]. These gendered and sexual identities are not just passively absorbed, but also actively re-enacted. Young women in the rural Eastern Cape province activate diverse femininities and foreground their agency in pursuit of their own sexual and emotional agendas, while at times accepting violence and patriarchal norms in return for economic and emotional benefits in the face of enduring poverty [19]. Furthermore, while young women’s agency was most notable at the start or initiation of relationships, this agency was constrained once relationships were more established, and patriarchal roles of male control were entrenched [20]. The authors document a diverse and dynamic set of femininities, ranging from a ‘modern girl’ femininity, a dominant conservative cultural model, as well as a blend of an emerging feminist consciousness, albeit still blended with a more traditional femininity. However, none of these included a significant challenge to the dominant gender arrangements, that are patriarchal and heteronormative. Strikingly, femininities constructed by young women, some of which invoked the discourse of ‘empowerment’, do not fundamentally destabilise men’s power, but serve to legitimize and reinforce patriarchal power structures.

Interpersonal relationships are constructed by gendered socio-economic material and physical contexts (e.g. gendered ownership of space by young men) and related cultural practices as they are ‘scripted’ into the functioning and practices of relationships. These provide barriers for young women to negotiate safer sexual practices [21]. Bhana, (2017) similarly shows how adolescent ideas and experiences of love are located within the broader social, economic and gendered context which include, chronic unemployment, poverty and historical legacies of apartheid*.* Both these papers illustrate the need for more nuanced approaches when researching and analysing adolescent interpersonal relationships, as they illustrate how the social and structural determinants, such as poverty, intersecting with and compounded by gender inequality, are part of reproducing inequality and vulnerabilities to HIV and gender-based violence (GBV).

Further, illustrating the cross-cutting determinants of the conceptual framework beyond heteronormative identities, learners in school who identify as LGBTIQA+ are often marginalised or bullied, ranging from derogatory language to violence, perpetrated by both learners and teachers [22]. These deeply patriarchal and heteronormative contexts have rendered queer learners ‘invisible’, compounded by racial and other inequalities, in constructing their experiences in the South African education system. Despite this, queer learners also resist dominant narratives, reflecting resistance and agency in trying to resist homophobia by developing counter narratives. Addressing heteronormativity as a social and structural determinant in school contexts will therefore require a more inclusive curriculum, informed by the experiences of queer learners and including counter-normative sexualities in the content and process of teaching sexualities education in South Africa [23].

Despite numerous interventions and substantial funding (e.g. DREAMS and the She Conquers Campaign) that has largely been directed to preventing HIV among adolescent girls and young women, gender relations remain firmly entrenched and reproduced by the dominant social and structural determinants, such as the intersections of patriarchy, racial and the geospatial legacy of apartheid, heteronormativity, poverty and class, for example [24, 25]. Within public health, many HIV and gender-based violence interventions in South Africa focus on norms experienced at the micro level and attempt to change individual behaviour related to sexual practices such as condom use, for example [26]. Moreover, in terms of retention in care and adherence to HIV treatment, there is need for interventions that go beyond health facilities, to address broader social and structural determinants at the meso and macro levels ([27, 28]). As illustrated by the conceptual framework and narrative examples summarised above, this alone is not enough. Further, interventions, including those which aim to be gender-transformative are often only partial, in that the individual behaviour they attempt to address, is shaped by and embedded in, the broader dynamic of social, cultural and interpersonal realms within structural contexts, that are left unaddressed [26, 29].

There is a need for expanded paradigms beyond just a focus on individual identities and vulnerabilities at the micro level, but to conceptualise how these are created and reproduced by the social and structural determinants at the meso and macro level, as well as the cross-cutting determinants and axes of inequality based on gender, race, class and sexual orientation. This is one of the key threads of our analysis and central to the conceptual framework we present.

Having noted the need for different paradigms and approaches it is important to highlight that globally there has been work done related to gender and economic interventions and addressing poverty as a central approach to preventing GBV and HIV. However, there is a need for more research looking specifically at adolescents, including those living in South Africa [30]. There is also a growing body of research on the role of adolescent-sensitive social protection, mostly focussed on HIV positive adolescents, as an approach to address both immediate and distal determinants, beyond just the micro level [27, 28].

### Meso level: organisational and sectoral

The meso level determinants described in this section focus on the organisational and sectoral as institutions and sites that determine adolescent health, with a focus on access to health care, education, school safety and sexualities education, as illustrative examples of the conceptual framework (Fig. 1). While recognising the importance of access to infrastructure and services such as transport, water and sanitation, there is a paucity of adolescent-specific data and research on these sectors. Therefore, they are not discussed further in this section and should be the focus of further research.

South Africa has enacted comprehensive and progressive legislation and policy frameworks to enable provision of and access to adolescent and youth friendly health services through the initial National Adolescent Friendly Clinic Initiative (NAFCI) and subsequent Adolescent and Youth Friendly Services (AYFS) [31]. Despite these investments adolescents report dissatisfaction with the quality of care received, in terms of respect and confidentiality, long waiting times and stockouts of medicines, as well as attitudes of health workers [32–34], as organisational determinants that shape their health. The AYFS  is aligned to The Ideal Clinic Initiative, a process of standard setting and quality improvement, which includes measures towards adolescent and youth friendly programmes. These include access and availability of adolescent services, relevant information and communication and a management system to support service delivery [35]. As this is a relatively new approach, further research is required to assess its impact on health systems responsiveness to diverse adolescents’ needs and experiences.

Within these initiatives to improve access of services to adolescents, interactions between adolescents and health workers are critical. Nurses providing sexual and reproductive health services to adolescents navigate conflicting roles as service providers, educators and law enforcers. In making decisions and ideological judgments about adolescents seeking services, they create barriers and differential access to services [36]. Apart from prejudicial attitudes and responses of providers, as well as lack of knowledge on sexual and reproductive health and rights by service providers, adolescents are also constrained by a lack of physical access to health facilities, limited range of contraceptives, as well as programmes that do not include adolescents and young people in their design and delivery [34, 37].

In addition, there are several additional barriers to sexual and reproductive health information and services for sexual and gender minority adolescents, which include stigma related to their sexual orientation, further compounded by age-related stigma in terms of being sexually active as adolescents [38]. Furthermore, adolescents living with disabilities also experience additional barriers to services and education, thus increasing their vulnerability to issues such as HIV and sexual violence [39].

The meso level also looks at the role of other sectors, and we review research on the education sector, and how it can create systems that promote, sustain or harm the health of adolescents, as illustrated in the conceptual framework. In South Africa, schooling is compulsory to the age of 15 and a no fee schools policy removes financial barriers to access in principle. However, for many children their school experience is marked by irregular attendance, absent teachers, teenage pregnancy and school-related abuse and violence. Despite huge strides being made in terms of access to education, persistent racial, geographic, socio-economic and gender inequalities shape access to and quality of education [40]. Around 27% of public schools do not have running water, 78% are without libraries and 78% do not have computers.

Adolescent girls face multiple additional barriers to education and specific risks to their health once in school. The use of poor quality sanitation in schools or avoidance due to their poor quality results in poor menstrual hygiene for adolescent girls [41]. In addition, the experience of sexual violence, from both male teachers and students, prevails as part of a broader school environment that is not safe and healthy for girls. While the national education policy is committed to gender equality and ensuring access to education for all learners, including those who may be pregnant or parents, in practice this is not the case. Macro level determinants, such as broader societal narratives and dominant ideas on teenage pregnancy, parenting and also female sexuality, also shaped by the power relations between school management teams, educators and learners, determine how this is addressed at schools [42]. These dominant discourses make schools a place of exclusion and unwelcoming of learners who are pregnant, by perpetuating normative gender roles and framing female sexuality and teenage pregnancy as social and moral degeneration.

We further illustrate the conceptual framework to show how the meso level determinants intersect with macro level and cross-cutting determinants, with the example of the provision of sexualities education. Life Orientation sexualities curriculum bombard learners with messages of ‘disease, danger and damage’ [43], unknowingly promoting rigid versions of gender, some of which underlie sexual violence, as well heteronormativity as macro level determinants of adolescent experience and health. Dominant notions of masculinity and femininity also include holding young women accountable for upholding both their own and societal moral practice, for example by placing the burden of preventing pregnancy, HIV and rape largely on them [44].

While it is important for adolescents to understand the contextual factors and risks related to sexual relationships, the dominant construction of young women as inevitable victims, needing protection and needing to self-control and restrain, is problematic for working towards gender justice. In addition, educators feel under-prepared and under-supported in teaching sexualities education and in providing support to learners who approach them with personal issues. Educators who themselves embody and perpetuate dominant gender norms, are not neutral, and are part of organisational determinants that reinforce gender inequality by what and how they teach i.e. largely didactic methods reinforcing narratives of illness and disease and girls needing to take responsibility [45].

Research comparing Life Orientation manuals with the songs voted most popular by these students highlight the contradictory pressures they face [46]. The findings illustrate the gaps between what adolescents are required to learn i.e. being responsible sexual subjects and danger of sexual victimisation, as opposed to what they are engaging with in their free time i.e. songs about sexual pleasure and tensions in relationships. In addition, the subjective experiences and what adolescents are wanting to discuss, is not addressed at school, however this is important for comprehensive sexualities education programmes in and out of school [47] .

Despite global evidence and national support, progress has been slow to develop and implement comprehensive sexualities education that includes a positive understanding of sexualities and gender and which creates a safe and non-judgemental space to meaningfully engage adolescents themselves. The current pedagogy is largely ‘expert’-based and didactic missing out on the critical space to actively engage adolescents in making meaning of gender and sexualities [48]. A recent development is that the Department of Basic Education is planning to roll out an updated Comprehensive Sexualities Education programme in 2020, and this is already facing steep resistance from religious and some parent groups [49, 50]. Despite the furore, the new curriculum provides a significant opportunity to support adolescents to build an accurate understanding of their bodies and develop healthy attitudes and behaviours when it comes to sexualities, identities and relationships and begin to address some of the cross-cutting social and structural determinants, such as gender inequality, gender-based violence and homophobia, for example.

This meso level focus highlights how health services and schools are not neutral spaces, but are shaped by the social and structural determinants and are highly gendered organisations, that both reflect and replicate notions of masculinities and femininities that are present in the broader social and political national and global contexts, as cross-cutting determinants. Further, it shows that the rights to health and education and non-discrimination described in policies, evaporate in organisations where adolescents, providers and management hold and reinforce regressive views and assumptions related to gender and related social and structural determinants, such as race, sexual orientation and class, for example.

### Macro level: institutional, structural, environmental and geospatial

As foregrounded in the conceptual framework (Fig. 1), the macro level determinants illustrated in this section include the institutional i.e. national laws and policies that govern the country and what the implications are for adolescent health. In addition there is a focus on the structural i.e. colonial and apartheid legacies, as well as current political and economic systems as well as environmental and geospatial components. It also includes broad outcomes and consequences of such legacies and systems, such as the nature of poverty and inequality, as well as violence and how these intersect with cross-cutting determinants. These macro level structural determinants related to education, employment, gender inequality and violence have only gotten worse with COVID-19 and they powerfully shape the experience of adolescent health.

Current inequality in South Africa has its roots in the historical influences of colonialism through the dispossession of land, migrant labour system and extraction of resources [51]. In addition, the apartheid system, articulated through legislation and structural racial inequalities, denial of democratic rights and violent state oppression, also deeply marked the nature of inequality. These historical legacies shaped power relations which are persistent post-1994. Key ‘fault lines’ in health system weaknesses remain including leadership, management and governance [52]. There is a disconnect between the progressive Constitution and the limited critical public discourse around the historical intersections of racial and gender inequality for example [53, 54]. The apartheid era policies created structural constraints, limiting possibility for social and economic opportunities and mobility for the vast majority of South Africans [55]. Despite political freedom in 1994, legacies of disadvantage remain and are reproduced across generations, i.e. intergenerational transmissions of economic and social capital, or lack thereof. Importantly, South Africa’s social and economic context is part of a broader system of global economic trends, which contributes to combined and uneven development, further perpetuating inequalities. This includes the nature of macroeconomic policy, economic regulation and the economy as a whole [56].

The historical context of colonialism, apartheid with its plethora of laws and policies that violated human rights, was interwoven with systems of patriarchy and complex constructions of masculinities through violence and gender hierarchies, all of which impact on the context in which adolescents are socialised and live [53, 54, 57]. Contemporary South Africa has some of the highest prevalence rates of violence against women and children, as well as HIV in the world, which are fuelled by gender and intersecting and compounding inequalities. Adolescent girls and young women in South Africa bear the brunt of HIV and GBV and these both illustrate how past and present macro level determinants dynamically interact, shaping the health of adolescents.

The rape and murder of a 19-year-old female student in August 2019 was a catalyst for national outrage and re-emerging protests against the continuing high rates of sexual violence and the persistent failure by government to reduce and stop gender-based violence, including femicide [58, 59]. These protests focussed the national debates again on the normalisation of gender-based violence including femicide and how this is perpetuated by a patriarchal and unequal society. While there is an increased awareness around gender-based violence, there is limited focus on problematising patriarchy as a social and structural determinant and how it constructs masculinities and femininities. With reference to the conceptual framework, it highlights the importance of the macro level determinants of gender based violence when conceptualising and planning interventions and services, addressing underlying causes and prioritising prevention, when allocating specific resources in the South Africa context [60].

A further illustration of the interaction between the social and structural determinants at macro, meso and micro levels are the many gangs, located in urban and peri-urban areas created by apartheid, which often operate as subcultures and an expression of, and resistance to, the dominant political systems. These physical and social spaces are also sites for creation of gendered identities which are layered with racial and economic and patriarchal power relations [61, 62]. In these spaces there is a complex dynamic between physical space, economics, gender and race, as adolescent boys and young men often have limited access to the key resources that define a dominant masculine identity and therefore use physical violence, murder and rape as an alternative means to assert their masculinities and personhood in these local contexts. Research conducted with young men in urban informal settlements highlights how diverse masculinities, often with disjuncture  to hegemonic masculinities, including hierarchy of masculinities, are expressed [63]. This research demonstrates how cross-cutting and intersectional axes of inequality in terms of gender, race, and class shape masculinities and endorses gender-transformative work, at the micro, meso and macro levels in order to address interrelated national priorties of HIV and GBV prevention, in the context of inclusive violence prevention.

The macro level also includes environmental and geospatial determinants that shape the physical and social spaces that adolescents occupy and are contemporary effects of the past intentional racial segregation, as noted in the conceptual framework. An illustration of this in post–apartheid South Africa is how these social and structural determinants continue to provide significant contextual challenges and barriers for adolescents in terms of access to education, employment and safe spaces to live and learn [64]. These findings suggest that spatial geographies of adolescents are significantly shaped by race and also gender, in that girls have ‘shrinking’ or curtailed spaces they access and very limited geographical spaces where they feel safe, whereas boys’ areas expand and contain a balance between safe and unsafe places. As per the conceptual framework, these findings illustrate that experiences of violence and the fear of violence, curtail the geographic spaces for adolescents and that this is a very gendered issue in that girls and boys manage their social and physical spaces very differently. The implications for adolescent health are important as we consider the relevance of creating safe spaces to access education and engage in productive and leisure activities as part of their growth and development into adulthood.

The interactions between macro, meso and micro levels determinants are also reflected in the narratives of lived experiences of everyday life for adolescents living in 3 proximal neighbourhoods in Cape Town, in terms of their relationships at school and home in a democratic South Africa, where apartheid ‘echoes’ in the continuing relationship of physical and social spaces being divided and unequal in terms of race and class as cross-cutting determinants [65]. The neighbourhoods are largely still organised along race and class and this has implications for how adolescents engage with the physical and social space, each other, as well as the exchange of ideas. In addition the cross-cutting determinants impact on experiences and narratives of adolescents’ in terms of relationships, highlighting the very binary stereotypes that dominate in terms of masculinities and femininities and the intersectionality of multiple forms of inequality. As notes by the authors, *“For boys who have few material resources and poor prospects, masculinity can become dependent on heterosexual success with girls, and even on violence and coercion. Girls in poor communities whose consumer aspirations do not fit with parental means of provision can become dependent on boyfriends, which can lead to submission in the face of sexual demands and acceptance of sexual coercion and violence (2010:292).”*

This builds on earlier work which describes the dynamics of racial and gendered identities in Manenberg in Cape Town, a township created by racial segregation, where adolescents and young people manage and mediate their identities as they interplay with global forces such as soap operas, rap music and international brand name clothes, which they make sense of through their local contextual realities [66, 67]. This body of work describes how the construction of adolescent sexuality, personhood and identity is shaped both by the apartheid history and articulated through the current social, geospatial and political contexts and speaks to the dynamics between global macro, meso and micro level determinants.

Another macro level determinant noted in the conceptual framework is the institutional i.e. the laws and policies that govern the country. In South Africa, we have a progressive Constitution and Bill of Rights, supported by national legal and policy frameworks, which are aligned to international conventions that seek to protect and promote human rights. Various key policies supporting adolescents and adolescent health include the National Youth Policy (2015), the National Adolescent and Youth Health Policy (2017), National Adolescent Sexual and Reproductive Health Rights Framework Strategy (2015), as well as two policies related to health in schools, Integrated School Health Policy (2012) and the National Policy on HIV, STIs and TB for learners, educators, school support staff and officials in primary and secondary schools in South Africa (2017).

Despite various policies in place, the institutional and regulatory framework around adolescent health is complex and contradictory at times and illustrates the interactions between macro and meso level determinants. For example, the legal framework on sexual and reproductive health for adolescents aims at providing access to services and promoting and protecting their rights, by providing access to contraception and termination of pregnancy from 12 years, while the Integrated School Health Policy allows children to access health services without consent from parents from 14 years. In addition the Sexual Offence and Amendment Act (2015), which sets the age of consent to sex at 16, also requires reporting by all services. This can create an environment which is infused with contradictions, confusing reporting requirements and differential access to services for adolescents [36] and create confusion and multiple roles for service providers as described in the meso level section. Of interest is the Standard Operating Procedures for the provision of Sexual and Reproductive Health, Rights and Social Services (2019), issued by the Department of Basic Education, which is a step towards policy alignment and more coordinated provision of sexual and reproductive health services. However, much work remains in implementing and realising the policy visions and scaling up initiatives, particularly those working across multiple sectors [68].

In summary, this section illustrates how the past and present macro level social and structural determinants have a significant impact on the meso and micro level determinants of adolescent health in South Africa, highlighting the critical role of broader contextual factors and systems that need to be considered. It raises critical issues in terms of needing to address and transform the past and present axes of inequality, such as sexism and racism, as central to our efforts to address the health and well-being of adolescents.

As shown in the conceptual framework, the South African context at micro, meso and macro levels is nested within a global context, however there is limited research on impact of these global social and structural determinants with specific focus on health of adolescents. Global level determinants that have an impact at national level include trade policies, social media and access to internet, neo-liberalism, ideologies, globalization, migration, war and conflict, climate change and planetary health, for example. South Africa’s social and economic context is part of a broader system of global economic which contributes to combined and uneven development and perpetuating inequalities [56]. The Lancet Commission on adolescent health and wellbeing (2016) highlighted the impact of global food, alcohol and tobacco polices as well as the role of social media on adolescent health [9].

A significant, but under-researched global level determinant is access to the internet and the use and experiences of digital resources targeted at providing adolescents with information about sexual and reproductive health and rights, contraception and education, as well as career guidance, however there is a paucity of empirical research for South African adolescents specifically. A recent study which reported on young people’s use of mobile phones for sexual and reproductive health and rights related activities identified nineteen services, none of which have been evaluated [69]. Going forward, given the COVID-19 pandemic, this remains an area for further content development based on best-practices and consultation with adolescents themselves as well as rigorous research.

**Key Messages**

**Table 1:** 

• Micro level: adolescent health is shaped by individual, interpersonal and community determinants. Adolescents exercise important autonomy and agency, however they do so within South African society that is still characterised by regressive norms and gender and intersecting power relationships and inequalities • Meso level: adolescent health is shaped by organisational and sectoral determinants and the health and education sectors provide important entry points for supporting the health and rights of adolescents, but they also can replicate the biases and inequalities that characterise South African society • Macro level: adolescent health continues to be shaped by the structural colonial and apartheid legacies and despite constitutional and other legislative rights, intersecting and compounding economic, geographic, gender and other inequalities, at national and global levels, shape adolescent health in democratic South Africa • Micro, meso and macro levels are also shaped by cross-cutting and intersectional social and structural determinants, and other forms of inequality and marginalisation based on gender, race, class, (dis) ability and sexual orientation, for example	

## Conclusions

Using a conceptual framework (Fig. 1) we describe and illustrate key social and structural determinants across interrelated and dynamic macro, meso and micro levels, as well as key cross-cutting and intersecting determinants, all of which dynamically interact with global determinants, to shape adolescent health in South Africa. In foregrounding the South African context, our analysis contributes to the international literature on adolescent health by demonstrating a systematic manner for moving beyond the micro level and also addressing social and structural determinants at the meso, macro and global levels [13, 14, 70–72]. A key message from our review is that current experiences and health of adolescents is shaped by past social and structural determinants and power relations, with apartheid inequalities still echoing in the lives of the ‘Born Frees’ [73]. Therefore, understanding both the historical context as well as contemporary social and structural determinants and intersecting and compounding power relations, provides significant insights into determinants of adolescent health beyond just the micro level, but also at the interrelated and dynamic meso and macro levels, nested in global determinants, as presented in our conceptual framework.

Our analysis shows the complexity of intersectional inequalities and how those at individual and interpersonal (micro) level are mediated through the institutional structures and organisational factors in health and other sectors (meso) level and how this is underpinned by the structural determinants such as the national and global political economy (macro) level. These are not linear relationships and gender and intersecting power relations are complex to change. They require careful analysis across macro, meso and micro levels, consultation with adolescents themselves and detailed research as part of understanding and transforming past and present social and structural determinants of adolescent health in South Africa. In addition, further work needs to build on insights gained from individual agency of adolescents, gender-transformative programme responses and implementation of progressive policy and legislative measures.

Going forward addressing the meso and macro level social and structural determinants, for example, poverty and youth unemployment, provision of quality education, improved alignment and implementation of laws and policies with and across departments, will contribute to transforming society in being more responsive to the rights and needs of adolescents, and in this way contribute to their health. Importantly, there is a need to strengthen and activate citizenry where adolescents can advocate for themselves and create and mobilise networks and organisations that raise critical issues and hold government and other actors accountable. Twenty-five years into democracy much work still remains to be done towards the health of adolescents centering their collaboration, in realising the rights enshrined in the Constitution and working towards the ambitious SGD goals and ensuring the principle of leaving no one behind.

## Data Availability

Data for this paper included publicly available articles and documents and no additional data was generated. These articles and documents can be made available should that be required.
